# Development and relationship between the judgment of the speed of passage of time and the judgment of duration in children

**DOI:** 10.3389/fpsyg.2023.1160047

**Published:** 2023-05-18

**Authors:** Natalia N. Martinelli, Sylvie Droit-Volet

**Affiliations:** Université Clermont Auvergne, CNRS, LAPSCO, Clermont-Ferrand, France

**Keywords:** time, passage of time, duration, children, consciousness, emotion, embodiment

## Abstract

This study examined the relationships between the awareness of the speed of the passage of time, the judgment of durations and experiential factors in children aged 4–9 years. They were asked to judge the duration and the speed of the passage of time for different intervals (second and minutes), and to rate their feelings (arousal, happiness, sadness, and task difficulty) during each interval. The results indicated that 8–9-year-olds’ judgment of the passage of time is extremely flexible and context-dependent, representing the duration and/or the individual changes in subjective experience (emotion). In contrast, young children’s judgment of the passage of time was not related to duration. However, their judgments were not given randomly. They judged that time passed more quickly when they felt happier and more alert. The passage-of-time judgment was therefore initially grounded in emotional and sensory-motor experience, i.e., in their perception of changes (acceleration and deceleration) in self-movement (successions of states and their extension). Therefore, duration judgment and passage-of-time judgment initially develop separately and are later combined when children understand the logical link between speed and duration.

## 1. Introduction

From early infancy, children can track the flow of time and learn durations of events or inter-event intervals. However, the fact that young children can perceive durations does not mean that they are aware of the passage of time. Adults express their awareness of the passage of time in terms of speed, for example, by stating that time passed quickly during that good movie and slowly during that boring, bad movie (Wearden, 2015; Droit-Volet and Martinelli, 2023). Thus, variations in the judgment of the passage of time in adults have usually been investigated by asking them questions such as “how quickly does time seem to pass, from very slowly to very quickly” (e.g., Larson and von Eye, 2006; Sucala et al., 2011; Droit-Volet and Wearden, 2015, 2016). According to experimental studies using this type of question, the feeling of a speeding up or slowing down of the passage of time depends on the non-temporal context and its subjective effect on the self (minimal self) (e.g., cognitive engagement, emotion, and difficulty of the task), rather than on the event duration *per se* (e.g., Larson and von Eye, 2006; Tipples, 2018; Cellini et al., 2020; Ogden, 2020; Witowska et al., 2020; Droit-Volet et al., in revision). Indeed, participants judge that time passes more quickly when viewing positive than negative emotional stimuli or when performing a difficult task compared to an easy one, although these stimuli and tasks are of similar duration (Martinelli and Droit-Volet, 2022a,b). The judgment of the passage of time is therefore dissociated from that of the objective amount of time (duration). Nevertheless, adults can also judge the passage of time of a given event duration when asked to do so in prospective temporal judgment tasks. They then report that time passes more quickly for short durations than long durations (i.e., small amount of time = fast passage of time) (Martinelli and Droit-Volet, 2022a,b). According to the contextual self-duration theory of the passage-of-time (Martinelli and Droit-Volet, 2022a,b; Droit-Volet and Martinelli, 2023), there are therefore multiple determinants of changes in the passage-of-time judgment, even though the emotion factor dominates. The aim of this pilot study is to investigate in children the relationships between the awareness of the speed of the passage of time, the judgment of durations and the main factors (emotion, task difficulty) identified as being related to the passage-of-time judgment in adults.

To be able to express this feeling about the speed of the passage of time, children need to be able to do four main things. Firstly, they must be able to differentiate between the judgment of the amount of time, i.e., the duration that has passed, on the one hand, and the self-duration judgment, on the other, i.e., say whether this time seems to them (from their perspective) to have passed slowly or quickly. Indeed, the feeling of the passage of time can vary for the same event duration (Martinelli and Droit-Volet, 2022a). The passage-of-time judgment depends on the introspective analysis of effects of context (emotion, task difficult) on changes in the self. Consequently, and secondly, they must be aware of their subjective experience, i.e., of changes in their internal states (phenomenological experience). Thirdly, as discussed later, they must understand the metaphor of motion and speed of motion in relation to time, i.e., a metaphor which is embedded in the spatialization of time: i.e., “time passing is motion” (Radden, 2011, p. 28). Fourthly, they must use this metaphor appropriately by associating a specific pace of time with a specific phenomenological experience, in the same way that adults do (e.g., more happiness equals faster time, more sadness equals slower time), or associate it with a specific stimulus duration (longer durations equal slower time). The judgment of the passage of time is therefore particularly complex because it calls on different cognitive processes, such as the acquisition of language and the ability to coordinate time with another dimension, i.e., speed.

To date, no studies have investigated either the development of the passage-of-time judgment or that of the relationship between this temporal judgment and the duration judgment. However, it has been shown that children are able to automatically process duration as of the first months of life (implicit judgment of duration) (e.g., Brannon et al., 2004; Provasi et al., 2011; de Hevia et al., 2014), and that, from the age of 3 years, they are able to correctly apply temporal instructions and estimate the duration of stimuli to be presented (explicit judgment of duration) (e.g., Droit-Volet and Wearden, 2001). Beyond this, the precision of explicit temporal judgment improves with the development of attention and working memory skills to achieve adult-like performance at 6 years for short durations (<1 s) and at 8–9 years for long durations (>1 s) (Hallez and Droit-Volet, 2020). As the passage-of-time judgment may be based on non-temporal information, we can assume that its developmental trajectory is initially independent of that of duration judgment. These two temporal judgments would only coincide perfectly when children understand the relationship between time and speed and pay attention to time (prospective time judgment). Therefore, we assume that before young children become capable of cross-dimensional comparisons, they should judge stimulus durations to be longer as their value increases. This would be consistent with their ability to discriminate durations. However, they should not be able to associate this duration judgment with a parallel slowing-down of the speed of time.

At 5–6 years, the speed at which moving objects move interferes with children’s judgment of their presentation duration, and they already have knowledge about the speed of “things” (e.g., slow speed for a tortoise, fast speed for a hare) (e.g., Matsuda, 1974; Hallez and Droit-Volet, 2018; Mioni et al., 2018). However, they do not grasp the complex relationship between time, speed, and space. Understanding the inverse ratio between time and speed (faster = shorter duration) requires inferential reasoning skills that appear late in childhood. By studying dynamic situations, Piagetian researchers long since demonstrated that it is not until around 8–9 years that children understand that the travel time of a fast-moving object is not necessarily longer because “faster = shorter duration” (Piaget, 1946; Levin, 1977; Montangero, 1977; Siegler and Richards, 1979). Before this key age, faster and further are often associated with a greater amount of time, following the rule “any more is more time” (Levin, 1982, p. 77). Therefore, we can assume that it would only be around the age of 8–9 years, when children can correctly coordinate speed and duration, that the judgment of the speed of the passage of time is systematically inversely proportional to the judgment of the duration, as it is in adults (“Fast speed = Shorter duration”; “Slow speed = Longer duration”).

Regarding the acquisition of spatial metaphors of time, several studies, mainly linguistic in nature, have examined how children refer to the past and future using a horizontal line with a front-back orientation (“ahead/behind”), refer to time as moving relative to an observer or vice versa [moving-time (“dinner is approaching”), moving-ego (“we are approaching the holidays”), or sequence-as-position (“summer follows spring”)] (e.g., Özçalışkan, 2005; McCormack and Hoerl, 2008, 2017; Stites and Özçalişkan, 2013; McCormack, 2015; Atance et al., 2019). However, to our knowledge, few developmental studies have addressed the question of the metaphor of changes in the speed of motion itself (time speeds up or slows down), even though the metaphors of motion and space are intertwined (Özçalışkan, 2007). The spatial metaphor for time consists in using a concrete concept (space) to represent a more abstract concept (time) which is not necessarily observable in the world (e.g., time which moves horizontally) (Boroditsky, 2000). However, it is debatable whether, for children, the motion metaphor for time (time passing) involves an abstract concept of time independent of the content of events, or whether it simply results from the intuition of motion that is perceived across the experience of individual changes in internal states or actions, i.e., relative to the minimal self (Droit-Volet and Dambrun, 2019). Indeed, rapid successions of different states and/or actions can be experienced in daily life as movements. Therefore, the sense of continuous time, involving both acceleration and deceleration, would derive from this sensation of movement and of changes in movement. The metaphor of the speed of time could thus initially be grounded in sensory-motor experience, as a sort of initial embodied “concept” of time (Stites and Özçalişkan, 2013). If this view is correct then, as explained above, young children would find it difficult to mentally associate variations in the speed of the passage of time with differences in duration. In other words, the metaphor of the speed of the passage of time would not, for them, represent time passing *per se*. Instead, it would represent perceived changes in their experience (see section “4. Discussion”). Consequently, we can assume that young children will state that time passes faster when they feel happier or when a task is difficult and, conversely, that it passes slower when they feel sadder, or when the task is easy.

This assumption nevertheless means that young children, at least at 4–5 years, are already able to use motion as a metaphor of time, even if it does not represent the concept of time independently of events. This is likely because, from the age of 4–5 years, they understand the metaphor of motion (e.g., “ideas wander through the mind”) and of moving time (“A trip to the zoo is approaching”) (Özçalışkan, 2005, 2007; Stites and Özçalişkan, 2013). However, if young children are not even capable of this type of early passage-of-time judgment grounded in subjective experience, then this judgment cannot be correlated with their level of subjective experience. They should either respond randomly or their responses should be inconsistent with those of adults, saying, for example, that time passes faster, and not slower, when they are sad.

The aim of our pilot study in this field of psychology was to investigate the relationships between the judgment of the speed of the passage of time, the judgment of durations, and experiential factors (arousal, happiness, sadness, task difficulty) in children aged from 4 to 9 years. Children’s judgments were assessed on several trials in a prospective time condition, in which the participants were asked to pay attention to time because they would subsequently have to estimate its duration. Children had to judge temporal intervals between two sounds in both the seconds and minutes range. We also used analog scales for these judgments like those used in other studies in young children (Friedman, 1990; Coughlin et al., 2014). In particular, Friedman (1990) demonstrated that by 4 years of age children successfully located familiar events on this type of scale on the basis of their relative duration. Our hypotheses were that, in a prospective duration judgment task, children from the age of 4–5 years would be able to make consistent judgments about the speed of the passage of time, but that these would be associated at a significant level with subjective experience but not with duration judgment. By contrast, in older children (8–9 years), they would be associated at a significant level with both subjective experience and duration judgment.

## 2. Materials and methods

### 2.1. Participants

The final sample consisted of 80 children aged from 4 to 9 years (38 girls and 42 boys), i.e., 34 children aged from 4 to 6 years (*M* = 4.5 years, *SD* = 0.52), and 46 aged from 7 to 9 years (*M* = 8.13, *SD* = 0.34). The young children were recruited from a nursery school and the older ones from a primary school, all in the Auvergne-Rhône-Alpes region of France. As a maximum of 3 factors were used in the regression models, we set a minimum of 30 participants per age group, as 10 observations per variable are generally recommended. There were no children with cognitive and affective impairments. Parents signed written informed consent to allow their children to participate in this study. The study was carried out according to the principles of the Helsinki Declaration and was approved by both the inspector of the Academy of the French National Education Ministry and the research ethics committee of Clermont Auvergne University (IRB00011540-2018-03).

### 2.2. Materials

The children were tested collectively in their classrooms during their morning school activities. The stimulus used to indicate the onset and offset of the interval duration to be judged was a sound produced by the experimenter striking a tambourine with a drumstick. The experimenter produced the stimuli by consulting a computer-clock system that indicated the inter-duration intervals and the interval durations. The experimenter could stop and restart this system in order to adapt to the class’s activities or events (e.g., break period). After each interval duration, the children gave their judgment on 6 different analog scales (paper-and-pencil version), all in the form of a horizontal line (19 cm) with a representative drawing at each end and a “verbal label” below: i.e., 6 for each type of judgment: duration, passage of time, arousal, happiness, sadness, task difficulty. They made their judgment by marking a vertical pencil line on the horizontal line (for a similar scale, see Friedman, 1990; Coughlin et al., 2014). For the duration judgment, there was a drawing of an empty hourglass (“very little time”) at the beginning of the scale and a drawing of a full hourglass (“a lot of time”) at the end (Friedman, 1990). For the passage-of-time judgment, there was a drawing of a tortoise (“very slowly”) at the beginning of the scale and a drawing of a hare (“very quickly”) at the end (Coughlin et al., 2014). For the judgment of emotions, we used scales similar to that used by Betella and Verschure (2016), for example, and they also proved to be similar to the Self-Assessment Manikin scale (SAM, Bradley and Lang, 1994), which is simple and practical for use in children from 3-1/2 to 14 years (e.g., Greenbaum et al., 1990; McManis et al., 2001; Sharp et al., 2006; Leventon and Bauer, 2016). For arousal, a quiet man (“calm, quiet, and asleep”) and an excited man (“irritated, agitated, awake”) were depicted at the beginning and the end of the scale, respectively. For the level of happiness and that of sadness, the children saw a neutral smiley (“not happy/sad at all”) and a smiling/sad smiley (“very happy/sad”). For task-difficulty, a character working effortlessly (“very easy”) or hard (“very difficult”) was depicted at either end of the sale.

### 2.3. Procedure

The children participated in two experimental sessions depending on the interval durations to be judged, i.e., in the seconds and minutes range. The session order was counterbalanced across subjects. The children completed 12 trials per session, with interval durations varying from 10 to 59 s and from 2 to 8 min. Specifically, there were 3 trials with target interval durations of 13, 27, and 53 s in the seconds condition, and three trials of target durations of 2, 4, and 8 min in the minutes condition, with 3 additional lure durations randomly selected in each temporal scale. The inter-trial intervals were also randomly chosen between 2 and 12 min. Due to class events, e.g., break times, which were longer for the younger children, we decided to spread the session over 1 day for the seconds condition and 2 days for the minutes condition for the older children, whereas the younger children performed the tasks on 2 days for the seconds and 3 days for the minutes condition.

Before the first session, children were presented with the six scales one by one. The experimenter and the teacher explained the meaning of each scale to them and told them that they could place the line wherever they wanted in the scale to indicate how they felt. A test trial using an interval duration of 15 s was then performed and the children responded on each scale. The experimenter explained to the children that they should not copy each other because there is no wrong or right answer, as this is very personal. It is their own feeling that matters. The experimenter also asked the children one by one about their responses to verify if the instructions had been understood. Another test trial was performed with an interval duration of 1 min and the responses were verified. Before each session, the experimenter reminded the children of the temporal task and the different judgments that they would have to make. The teacher then conducted the school day as usual. Before each trial, and without interrupting the class, the experimenter placed a booklet containing the scales next to each child seated at a table. Then, before each trial, the experimenter instructed the children to pay attention to the first and the second stimulus produced by the experimenter because they would have to judge the duration between these two stimuli. As it was difficult to resume the school activity between two stimuli (in particular for the seconds condition), and to make sure the children paid attention to the interval duration, we decided to instruct them to stop their school activity during each inter-stimulus interval and to wait and stay seated until they heard the second stimulus. The task during the interval to be judged was therefore easy but boring, thus making it possible to obtain differentiated answers for the different scales (e.g., sadness, difficulty, and duration). This also allowed us to check whether the young children always responded in the same way on the different scales (e.g., always on the right) whatever the judgment. After the second stimulus, the children therefore indicated their judgments on the six scales: duration and passage of time, arousal, happiness, sadness, and task-difficulty. The children’s responses on each scale were coded in cm (from 0 to 19 cm) from the beginning of the scale (horizontal line) to the child’s pencil line.

### 2.4. Data analyses

All statistical analyses were performed using SPSS software (Version 26, IBM Corp., Armonk, NY, United States) and Jamovi for the normality tests. As we used a series of six related scales for the same interval duration, a series of linear mixed model analyses (LMM) were performed with the duration judgment and the passage-of-time judgment as dependent variable. Subjects were always used as a random factor. Using these regression-based analyses, we first tested the effect of age, interval duration, and temporal range as fixed factors. Second, we analyzed the relationship between the duration judgment and the passage-of-time judgment. Third, we tested each experiential factors (arousal, happiness, sadness, and task-difficulty), for each age group and temporal range taken separately. The LMM is robust for non-parametric data (see Arnau et al., 2012). Nevertheless, we systematically verified the normality test using the Kolmogorov-Smirnov, as our sample was larger than 50 (Mishra et al., 2019). This was not significant for all analyses (all *p* > 0.05).

## 3. Results

**FIGURE 1:**
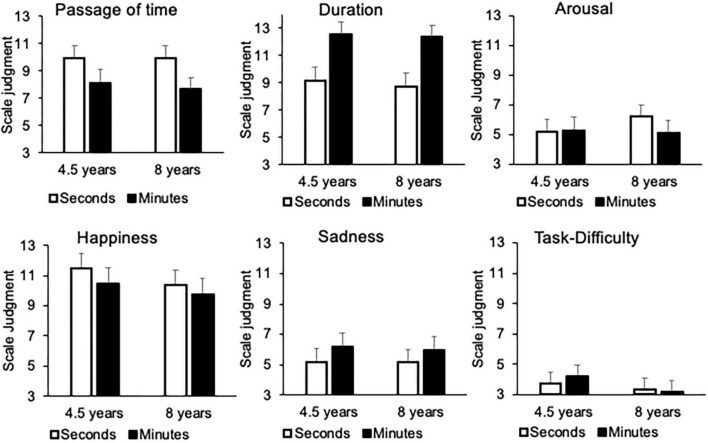
Average scores of both 4.5 and 8 years groups for: passage of time **(upper left panel)**, duration **(upper middle panel)**, arousal **(upper right panel)**, happiness **(lower left panel)**, sadness **(lower middle panel)**, and task-difficulty **(lower right panel)**.

The average judgments made by young (4.5 years) and older children (8 years) on the different scales are presented in Figure 1. This figure clearly shows that, from the age of 4–5 years, children gave differentiated responses for the different scales, responding, for example, toward the right of the scale for the emotion of happiness (e.g., 4.5 years, *M* = 10.95, ES = 0.95) and toward the left for that of sadness (*M* = 5.96, ES = 0.09), respectively. This demonstrates that they understood and used the scales correctly.

### 3.1. Duration judgment

**FIGURE 2:**
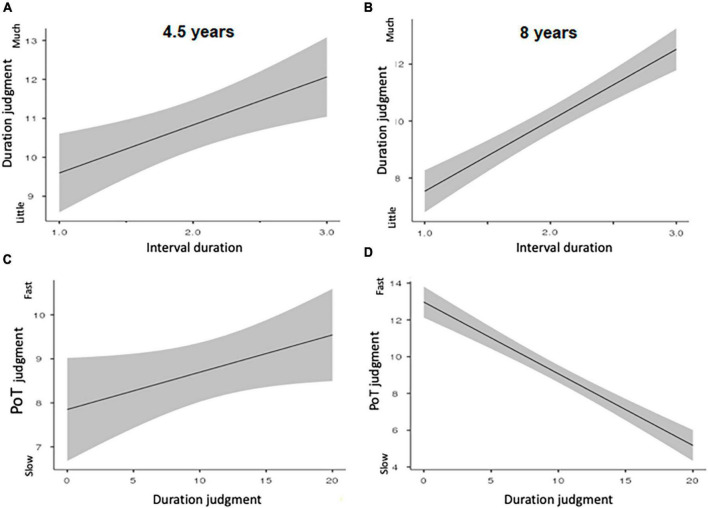
Mean duration judgments plotted against interval duration for 4.5 years **(A)** and for 8 years’ groups **(B)**. Mean passage of time judgments plotted against duration judgments for 4.5 years **(C)** and 8 years **(D)**.

An initial LMM was run with duration judgment as dependent variable and age, interval duration, and temporal range (seconds/minutes) as fixed factors. This LMM showed no effect of age, nor any effect involving the age factor (*p* > 0.05). Indeed, when we split the data on age group and temporal scale (seconds/minutes), the 5-year-olds appeared to be able to judge that the amount of time increased with the length of the interval duration for both the seconds (*E* = 1.38, *SE* = 0.41, 95% *CI* [0.56; 2.19], *t* = 3.35, *p* < 0.001) (Figure 2A), and the minutes durations (*E* = 0.89, *SE* = 0.41, 95% *CI* [0.85; 1.70], *t* = 2.18, *p* = 0.03). The minutes durations were also judged longer than the seconds durations (*E* = 10.84, *SE* = 0.81, 95% *CI* [9.20; 12.45], *t* = 13.46, *p* < 0.001) (Figure 1). Like the younger children, the 8-year-olds differentiated the length of the interval durations regardless of the duration range (seconds: *E* = 2.16, *SE* = 0.24, 95% *CI* [1.68; 2.64], *t* = 8.85, *p* < 0.0001; minutes: *E* = 2.82, *SE* = 0.30, 95% *CI* [2.23; 3.40], *t* = 9.47, *p* < 0.001) (Figure 2B), with the minutes being judged longer than the seconds (*E* = 3.14, *SE* = 0.41, 95% *CI* [2.34; 3.94], *t* = 7.70, *p* < 0.001).

### 3.2. Passage-of-time judgment

Contrary to the duration judgment, the LMM on the passage-of-time judgment with the same factors (age, interval duration and temporal range, and subjects as random factor) showed a significant 3-way interaction (*E* = −2.56, ES = 0.99, 95% *CI* [−4.16; −0.56], *t* = −2.52, *p* < 0.01), with the effect of age just failing to reach significance (*E* = −05.62, ES = 2.96, 95% *CI* [−11.43; 0.19], *t* = 1.90, *p* = 0.058). Indeed, there was a significant effect of interval duration on the passage-of-time judgment for the seconds (*E* = −1.97, *SE* = 0.57, 95% *CI* [−3.09; −0.84], *t* = −3.45, *p* < 0.001), but not for the minutes (*E* = −0.16, ES = 0.58, 95% *CI* [−1.29; 0.97], *t* = −0.28, *p* > 0.05) in the 4.5-year-olds (Table 1). By contrast, the effect of interval duration on the passage-of-time judgment was always significant in the 8-year-olds (Table 2), both for the seconds (*E* = −1.36, *SE* = 0.44, 95% *CI* [−2.23; −0.49], *t* = −3.07, *p* < 0.001), and the minutes (*E* = −2.05, *SE* = 0.42, 95% *CI* [−2.87; −1.23], *t* = −4.91, *p* < 0.0001). However, like the 8-year-olds, the 5-year-olds judged that time passed slower for the durations of several minutes than for those of a few seconds (Figure 1) (4.5 years, *E* = −1.81, *SE* = 0.67, 95% *CI* [−3.13; −0.49], *t* = −2.70, *p* < 0.01; 8 years, *E* = −2.67, *SE* = 0.51, 95% *CI* [−3.67; −1.68], *t* = −5.28, *p* < 0.0001).

**TABLE 1:** Potential predictors of passage-of-time judgments for the 4.5-year-olds in the seconds and the minutes condition.

	Estimate	Confidence interval	SE	*t*	*p*
**Seconds**					
Interval duration	−1.967	(−3.089; −0.845)	0.569	−3.45	<0.001
Arousal	0.199	(0.056; 0.341)	0.072	2.75	<0.01
Happiness	0.157	(0.029; 0.280)	0.064	2.43	<0.01
Sadness	0.082	(−0.070; 0.234)	0.077	1.07	>0.05
Task difficulty	0.238	(0.055; 0.421)	0.093	2.56	<0.01
Duration judgment	0.109	(−0.019; 0.237)	0.065	1.66	>0.05
**Minutes**					
Interval duration	−1.158	(−1.289; 0.973)	0.575	−0.28	>0.05
Arousal	0.267	(0.144; 0.390)	0.625	4.27	<0.0001
Happiness	0.182	(0.068; 0.297)	0.058	3.13	<0.001
Sadness	0.059	(−0.073; 0.191)	0.067	0.88	>0.05
Task difficulty	0.135	(−0.021; 0.292)	0.080	1.70	>0.05
Duration judgment	0.112	(−0.013; 0.236)	0.063	1.76	>0.05

The predictor is shown along with its associated estimate (coefficient), the confidence interval (± 95%), the standard errors. *t*-score and *p*-value.

**TABLE 2:** Potential predictors of passage-of-time judgments for the 8-year-olds in the seconds and the minutes condition.

	Estimate	Confidence interval	SE	*t*	*p*
**Seconds**					
Interval duration	−1.357	(−2.226; −0.488)	0.442	−3.07	<0.001
Arousal	−0.094	(−0.219; 0.030)	0.063	−1.49	>0.05
Happiness	0.086	(−0.013; 0.185)	0.050	1.71	>0.05
Sadness	−0.198	(−0.316; −0.082)	0.060	−3.33	<0.001
Task difficulty	−0.283	(−0.428; −0.128)	0.078	−3.59	<0.0001
Duration judgment	−0.358	(−0.457; −0.256)	0.051	−6.93	<0.0001
**Minutes**					
Interval duration	−2.053	(−2.873; −1.231)	0.418	−4.91	<0.0001
Arousal	0.128	(0.002; 0.254)	0.064	1.99	>0.05
Happiness	0.140	(0.044; 0.236)	0.049	2.87	<0.01
Sadness	0.045	(−0.062; 0.153)	0.055	0.83	>0.05
Task difficulty	0.146	(−0.007; 0.301)	0.078	1.87	>0.05
Duration judgment	−0.369	(−0.472; −0.267)	0.052	−7.03	<0.0001

The predictor is shown along with its associated estimate (coefficient), the confidence interval (± 95%), the standard errors. *t*-score, and *p*-value.

### 3.3. Relationship between passage-of-time judgment and duration judgment

The relationship between the passage-of-time judgment and the duration judgment did not reach significance in the 4.5-year-olds for either the seconds or for the minutes (both *p* > 0.05, Table 1). However, when we included the seconds and the minutes durations in the same model but without differentiating between them and also included the interval duration and the duration judgment as factors, we observed that when the duration was judged longer, the 4.5-year-olds did not tend to judge that time passed slower, but faster (*E* = 0.15, *SE* = 0.06, 95% *CI* [0.02; 0.28], *t* = 2.33, *p* < 0.05). As illustrated in Figure 2C, the longer the duration was judged to be, the faster time was judged to pass.

Unlike in the 4.5-year-olds, the relationship between the judgment of the passage of time and the judgment of duration was always significant for the 8-year-olds. Indeed, time was judged to pass slower the longer the duration was judged to be, and this for both the seconds and the minutes (*E* = −0.36, *SE* = 0.05, 95% *CI* [−0.46; −0.26], *t* = −6.93, *p* < 0.0001; E = −0.37, *SE* = 0.05, 95% *CI* [−0.47; −0.27], *t* = −7.03, *p* < 0.0001, respectively) (Figure 2D).

### 3.4. Relationships between passage-of-time judgment and experiential factors (arousal, happiness, sadness, and task-difficulty)

At the age of 4.5 years, the passage-of-time judgment was not related to duration judgment but to the subjective feeling experienced during the interval to be estimated. For the short durations (seconds), the 4.5 -year-olds did indeed report a speeding-up of time when they felt happier (*E* = 0.16, *SE* = 0.06, 95% *CI* [0.029; 0.28], *t* = 2.43, *p* < 0.01), and when their arousal level increased (*E* = 0.20, *SE* = 0.07, 95% *CI* [0.056; 0.34], *t* = 2.75, *p* < 0.01) (Table 1). They also judged that time passed faster when the task was judged as being more difficult (*E* = 0.24, *SE* = 0.09, 95% *CI* [0.55; 0.42], *t* = 2.56, *p* < 0.01). In the youngest children, sadness was not a significant predictor of passage of time (*E* = 0.08, *SE* = 0.08, 95% *CI* [−0.07; 0.23], *t* = 1.07, *p* > 0.05). However, when the three significant factors (happiness, arousal, and task difficulty) were entered in the same model, it was the level of happiness that was found to be the main predictive factor (*E* = 0.28, *SE* = 0.097, 95% *CI* [0.14; 0.42], *t* = 3.94, *p* < 0.0001), although the other factors did not lose their predictive power (*p* < 0.01).

**FIGURE 3:**
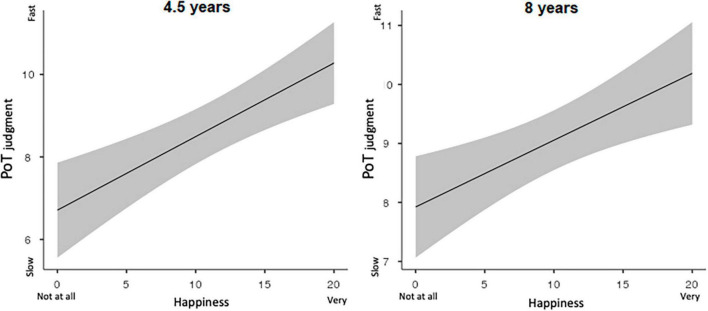
Mean passage of time judgments plotted against happiness rates for 4.5 years **(left panel)** and for 8 years’ groups **(right panel)**.

For the long durations (minutes), only happiness and arousal level were found to be significant predictors of the passage-of-time judgment in the 4.5-year-old children (*E* = 0.18, *SE* = 0.06, 95% *CI* [0.07; 0.30], *t* = 3.13, *p* < 0.001; *E* = 0.27, *SE* = 0.06, 95% *CI* [0.14; 0.39], *t* = 4.27, *p* < 0.0001, respectively). The level of sadness and task difficulty did not reach significance (both *p* > 0.05). This indicates that the 4.5-year-olds systematically reported a speeding-up of time when they felt happier (Figure 3).

For the 8-year-olds, the emotion-related factors were again significant predictors of the passage-of-time judgment. For the seconds range, sadness was the most significant predictor of the passage-of-time judgment (*E* = −0.20, *SE* = 0.06, 95% *CI* [−0.32; −0.08], *t* = −3.33, *p* < 0.001), while happiness and arousal did not reach significance (*E* = 0.09, *SE* = 0.05, 95% *CI* [−0.01; 0.185], *t* = 1.71, *p* > 0.05; *E* = −0.09, *SE* = 0.06, 95% *CI* [−0.22; 0.03], *t* = −1.49, respectively, both *p* > 0.05).

For the minutes range, the most significant predictor in the 8-year-olds was happiness, with children reporting a speeding-up of time when they felt happier (*E* = 0.14, *SE* = 0.05, 95% *CI* [0.044; 0.236], *t* = 2.87, *p* < 0.01) (Figure 3). The other factors (arousal, sadness, task difficulty) did not reach significance (*p* > 0.05). Task difficulty was also a predictor of the passage-of-time judgment, but only for the seconds duration (*E* = −0.28, SE = 0.08, 95% *CI* [−0.43; −0.13], *t* = −3.59, *p* < 0.0001). However, unlike the 4.5-year-olds, who reported a speeding-up of time when the task was perceived as being more difficult, the 8-year-olds reported a slowdown of time.

Like the 4.5-year-olds, the 8-year-olds therefore specifically experienced a speeding-up of time when they felt happier (Figure 3). However, in the prospective temporal judgment task used in the present study, the interval duration values continued to be the main predictor of the 8-year-olds’ passage-of-time judgment for both the short durations (*E* = −1.39, *SE* = 0.44, 95% *CI* [−2.25; −0.52], *t* = −3.12, *p* < 0.01), and the longer durations (*E* = −2.01, *SE* = 0.42, 95% *CI* [−2.83; −1.18], *t* = −4.78, *p* < 0.0001) when all significant factors were included in the same model. This finding is in contrast to the results obtained for the 4.5-year-olds. However, the other factors remained significant, although to a lesser extent (seconds: sadness, *E* = −0.14, *SE* = 0.07, 95% *CI* [−0.27; −0.01], *t* = −2.18, *p* < 0.01, task difficulty, *E* = −0.18, *SE* = 0.09, 95% *CI* [−0.36; −0.01], *t* = −2.06, *p* < 0.01; minutes: happiness, *E* = 0.13, *SE* = 0.47, 95% *CI* [0.03; 0.22], *t* = 2.69, *p* < 0.01).

## 4. Discussion

The aim of our psychology study was to investigate the relationships between the judgment of the speed of the passage of time, the judgment of durations, and experiential factors (arousal, happiness, sadness, and task-difficulty) in children aged from 4 to 9 years.

Our results showed that older children aged around 8–9 years produced temporal judgments similar to those found in studies in adults. Indeed, in our study, the 8–9-year-olds were able to discriminate different durations in both the seconds and minutes ranges. In addition, their judgments of the passage of time were consistent. Indeed, like adults, they judged that time passed more slowly as the objective value of the durations (interval duration) increased and as their feeling that the durations were becoming longer grew (duration judgment). Like adults, they also experienced a slowing-down of the passage of time when they felt sadder and a speeding-up when they felt happier (e.g., Droit-Volet and Wearden, 2015; Droit-Volet et al., 2021; Martinelli et al., 2021; Loose et al., 2022; Ogden and Piovesan, 2022). However, in the prospective temporal judgment condition used in our study, the objective interval duration remained the best predictor of their passage-of-time judgment. This finding provides support for the contextual theory of the passage-of-time judgment (Martinelli and Droit-Volet, 2022a) according to which this judgment is based on multiple factors (temporal or non-temporal) depending on context. It will nevertheless be interesting to further examine children’s passage-of-time judgments in a retrospective time judgment condition in order to identify whether certain contextual factors are favored by children compared to adults.

Nevertheless, our results suggest that 8–9-year-olds’ judgment of the passage of time is extremely flexible and represents both the flow of time and/or the individual changes in subjective experience (e.g., emotion). At these ages, this flexibility in judging the passage of time may result not only from the fact that children’s cognitive capacities (executive functions, inferential reasoning, and cross-dimension comparisons) are now sufficiently developed, but also from their knowledge about time: mastery of the concept of time (McCormack, 2015), mastery of spatial metaphors of time, including that of motion (Özçalışkan, 2005, 2007), and mastery of the links between time, speed and space (distance) (S = D/T; *D* = S/T, *T* = D/S) (Piaget, 1946). They therefore understand the complexity of time as it is conceived by humans, which can be both variable in phenomenological experience and constant in its measurement. By the age of 8–9, children thus process time and also think about it within the context of their cultural standards (Hoerl and McCormack, 2019).

Numerous studies have shown that, like 8-year-olds, younger children aged 4–5 years can process time (duration) even if their estimates are less precise. Our results using analogical temporal scales replicated this finding by showing that these children are able to discriminate different interval durations in both the range of seconds and that of minutes. However, unlike the 8-year-olds, they encountered difficulties when asked to judge the passage of time. In our study, they were not able to mentally associate an increase in the interval durations with a slowing-down of the passage of time (longer interval = slower passage of time) (at least for the long durations of several minutes), or to associate a feeling of the slowing-down of the passage of time with the feeling of lengthening of time (duration judgment). Indeed, no significant relationship between passage-of-time judgment and duration judgment was found in the younger children for either the short or the long durations. As our results suggest, these difficulties do not mean that they were unable to estimate different durations and speeds, but rather that they were unable to mentally coordinate these two dimensions. This difficulty in coordinating duration and speed needs to be verified in other contexts, such as individual rather than group administration as used in our experiment, although children’s responses were consistent even in this collective condition.

In addition, the difficulties experienced by children of this age in understanding the inverse ratio between time and speed have been well documented in Piagetian studies. Several studies have found that young children did not correctly infer time from other dimensions. When time co-varies with a non-temporal dimension (e.g., speed, number, and intensity), their time judgment is based on this other dimension. A moving object that goes further because it moves faster is, for example, judged to take more time. A light that shines more brightly is deemed to last longer. As explained by Levin (1982), children of this age seem to apply the rule “more of = more time” (see also Montangero, 1977). Our study provides results consistent with this statement. When we aggregated judgments for the short and long durations, the young children tended to judge the passage of time as being not slower but faster as their duration judgments increased. This error in coordination between time and another dimension has been explained in terms of interfering processes which are due to young children’s limited attention capacities. These interferences prevent them from ignoring non-temporal information which is more salient than the concurrent temporal information. In our study on the relationship between judgments of the duration and the speed of the passage of time, we can also assume that lack of knowledge regarding the concept of time and its links to space and speed also plays a key role. Indeed, a major shift occurs in children’s thinking around the age of 4–5 years (McCormack and Hoerl, 2017). Further experiments are nevertheless needed to examine how the acquisition of explicit knowledge about time modifies temporal judgments. In addition, the lower working memory capacities of young children could possibly have an impact on the link between the different temporal judgments examined in our study, because such children might have had problems remembering their previous time judgments and, consequently, the links between them. This also needs to be verified experimentally.

Even if the speed of the passage of time was not correctly related to duration judgment in the younger children, our results revealed that their judgments were not given randomly. From the age of 4 to 5 years, they did indeed judge time to pass faster when they felt happier and more alert. They therefore possess an awareness of the speed of the passage of time. However, this awareness is not related to changes in the flow of time and does not represent the passing of time. Instead, it relates to changes in the children’s internal state (emotion and arousal). This supports the idea that motion metaphors of time result from the embodiment of time (Özçalışkan, 2005, 2007). Some authors have considered that the spatial metaphor and its sub metaphors related to motion consist of using the more concrete concept of space to represent the more abstract concept of time, which is difficult to understand (Boroditsky, 2000). However, our finding with young children suggests that, initially, it literally reflects what children experience in everyday life: i.e., changes (acceleration and deceleration) in self-movement, specifically in successions of states and their extension. The idea of a continuous time with acceleration and deceleration would thus derive from this feeling of movement. A whole series of evidence suggests that sensory-motor experience helps children to construct an explicit representation of time (Coull and Droit-Volet, 2018). For example, younger children (3–4 years) make more accurate explicit temporal judgments when the duration to be judged is filled by an action performed by them than when the duration is empty (Droit-Volet, 2008), and also when they receive a force instruction (press hard) than a duration instruction (press longer) (Droit-Volet, 1998). In addition, motor imitation instructions (Droit, 1995) or motor training (Monier et al., 2019) promote the representation of time in memory. Action therefore helps children to feel motion, i.e., the changes in the succession of information. This would therefore provide the premise for the idea of a continuous time which sometimes accelerates and decelerates. However, in our study, we tested the emotional predictors of the passage-of-time judgment but not the motor predictor. The two factors are certainly linked in experience because differences in emotional and arousal states involve differences in motor energy. Nevertheless, the role of action and that of emotion need to be better differentiated in further studies on the passage-of-time judgment in order to determine which factor prevails in children compared to adults.

Overall, our results lead us to propose a developmental version of the Contextual self-duration theory of the passage-of-time judgment (Martinelli and Droit-Volet, 2022a,b). According to this developmental theory, the duration judgment and the passage-of-time judgment would initially develop separately, with the latter being based on self-conscious internal states. At this age, the feeling of happiness would appear to be the most important factor, with additional factors presumably emerging with increasing age. Later, when the concept of a time that is independent of events emerges, the passage-of-time judgment and the duration judgment become related in prospective time judgment tasks. The passage of time would be judged to be slower for long durations of several minutes than for short durations of a few seconds. However, for durations in the same temporal scale (where the contrast is less marked subjectively), young children would still fail to differentiate between the speeds of the passage of time for different durations, and/or would tend to apply the rule “more time = faster passage of time.” It would only be from the age of 8 years that children apply the opposite rule “more time = slower passage of time” for all duration ranges. This latter rule does indeed require children to understand the logical link between speed and duration. In conclusion, our study suggests that the feeling of the passage of time is already present at around the age of 4 years. At this age, however, it is grounded in emotional and sensory-motor experience and only later starts to represent the passage of time.

## Data availability statement

The raw data supporting the conclusions of this article will be made available by the authors, upon reasonable request.

## Ethics statement

The studies involving human participants were reviewed and approved by the Research Ethics Committee of Clermont Auvergne University (IRB00011540-2018-03). Written informed consent to participate in this study was provided by the participants’ legal guardian/next of kin.

## Author contributions

NM collected the data from participants in schools. Both authors contributed to the article and approved the submitted version.
